# High-Throughput Genetic Screen Reveals that Early Attachment and Biofilm Formation Are Necessary for Full Pyoverdine Production by *Pseudomonas aeruginosa*

**DOI:** 10.3389/fmicb.2017.01707

**Published:** 2017-09-05

**Authors:** Donghoon Kang, Natalia V. Kirienko

**Affiliations:** Department of Biosciences, Rice University Houston, TX, United States

**Keywords:** *Pseudomonas aeruginosa*, biofilm, pyoverdine, *Caenorhabditis elegans*, c-di-GMP, high-throughput screen, biofilm inhibitors, anti-virulence

## Abstract

*Pseudomonas aeruginosa* is a re-emerging, multidrug-resistant, opportunistic pathogen that threatens the lives of immunocompromised patients, patients with cystic fibrosis, and those in critical care units. One of the most important virulence factors in this pathogen is the siderophore pyoverdine. Pyoverdine serves several critical roles during infection. Due to its extremely high affinity for ferric iron, pyoverdine gives the pathogen a significant advantage over the host in their competition for iron. In addition, pyoverdine can regulate the production of multiple bacterial virulence factors and perturb host mitochondrial homeostasis. Inhibition of pyoverdine biosynthesis decreases *P. aeruginosa* pathogenicity in multiple host models. To better understand the regulation of pyoverdine production, we developed a high-throughput genetic screen that uses the innate fluorescence of pyoverdine to identify genes necessary for its biosynthesis. A substantial number of hits showing severe impairment of pyoverdine production were in genes responsible for early attachment and biofilm formation. In addition to genetic disruption of biofilm, both physical and chemical perturbations also attenuated pyoverdine production. This regulatory relationship between pyoverdine and biofilm is particularly significant in the context of *P. aeruginosa* multidrug resistance, where the formation of biofilm is a key mechanism preventing access to antimicrobials and the immune system. Furthermore, we demonstrate that the biofilm inhibitor 2-amino-5,6-dimethylbenzimidazole effectively attenuates pyoverdine production and rescues *Caenorhabditis elegans* from *P. aeruginosa*-mediated pathogenesis. Our findings suggest that targeting biofilm formation in *P. aeruginosa* infections may have multiple therapeutic benefits and that employing an unbiased, systems biology-based approach may be useful for understanding the regulation of specific virulence factors and identifying novel anti-virulence therapeutics or new applications for existing therapies for *P. aeruginosa* infections.

## Introduction

Antibiotic resistance is a catastrophic, re-emerging threat to health care. Multidrug-resistant nosocomial infections increase the danger to hospitalized patients and drastically increase healthcare costs (Brusselaers et al., 2011). Despite the fact that multi-drug resistant *P. aeruginosa* is already responsible for more than 13% of hospital-acquired *P. aeruginosa* infections, poor understanding of *P. aeruginosa* pathogenesis has limited viable treatments to conventional antimicrobials (Centers for Disease Control and Resistance, 2013). This is problematic for several reasons. First, *P. aerguinosa* forms antibiotic resistant biofilms that are extremely difficult to disperse and that limit penetration of drugs or the immune system. Second, *P. aeruginosa* expresses a number of multidrug efflux pumps, which further reduce intracellular antibiotic concentrations (Lomovskaya et al., 2001). Third, the pathogen readily acquires additional resistance mechanisms from other microbes. These phenomena make it imperative that we develop new treatments, beyond conventional antimicrobials.

The most promising alternative approach for limiting *P. aeruginosa* infection is to identify mechanisms to limit its virulence, rather than its growth. This target is complicated however, as mechanisms underlying pathogenesis vary widely, depending on infection conditions and environmental factors. A short, and incomplete, list of virulence factors encoded by *P. aeruginosa* includes cyanide- and phenazine-based toxins, type three secretion effectors, phospholipases, and exotoxins (Meyers and Berk, 1990; Gallagher and Manoil, 2001; Diaz and Hauser, 2010). *P. aeruginosa* also secretes the siderophore pyoverdine, which is necessary for full pathogenesis in various mammalian and non-mammalian hosts (Takase et al., 2000; Imperi et al., 2013; Kirienko et al., 2013; Lopez-Medina et al., 2015; Minandri et al., 2016).

The reason for this requirement remains unclear, but has generally been attributed to two different activities of pyoverdine. First, pyoverdine exhibits extremely high affinity to ferric iron (10^32^ M^−1^), which is sufficient to remove iron from mammalian iron-sequestering proteins such as lactoferrin and transferrin (Meyer et al., 1996; Xiao and Kisaalita, 1997). Iron is required for all life, and is extremely restricted within the host; pyoverdine is likely to help meet this demand. Beyond its role in scavenging iron, pyoverdine is also known to regulate the production of multiple other pathogenic determinants, such as the translational inhibitor exotoxin A and the protease PrpL (Lamont et al., 2002).

A recently established *Caenorhabditis elegans*-based pathogenesis model suggests an additional function for pyoverdine in virulence: it directly exerts cytotoxicity by removing host iron, inducing mitochondrial damage, mitophagy, and a lethal hypoxic crisis in the host (Kirienko et al., 2013, 2015). Multiple transcriptome analyses indicate that pyoverdine may also be involved in other aspects of *P. aeruginosa* pathogenesis, including quorum sensing and responding to reactive oxygen species (ROS) (Palma et al., 2004; Dietrich et al., 2006). Furthermore, small molecule pyoverdine biosynthesis inhibitors, such as 5-fluorocytosine and 5-fluorouridine are sufficient to rescue mice and *C. elegans* (Imperi et al., 2013; Costabile et al., 2016; Kirienko et al., 2016). This demonstrates the utility of limiting pyoverdine production in *P. aeruginosa* infections.

In large part due to its significance in virulence, a substantial body of work on the regulation of pyoverdine biosynthesis exists. The most directly relevant factor is the alternative sigma factor PvdS, which is regulated at both the transcriptional and post-transcriptional levels (Cunliffe et al., 1995). At the transcriptional level, *pvdS* expression is regulated by several factors, including intracellular iron concentration via the ferric uptake regulator (FUR) (Ochsner et al., 1995). Recently, the LysR-type transcriptional regulators CysB, OxyR, and PA2206 have been show to regulate *pvdS* expression under conditions of iron deficiency (former) or oxidative stress (latter two) (Imperi et al., 2010; Wei et al., 2012; Reen et al., 2013; Llamas et al., 2014). Post-transcriptionally, PvdS activity is controlled by FpvR, which sequesters PvdS to the inner leaflet of the plasma membrane (Edgar et al., 2014). PvdS's transcriptional control is also influenced by the regulator AlgQ, which helps to recruit RpoD (Ambrosi et al., 2005). AlgQ is well-known for its role in activating alginate biosynthesis, which plays a critical role during chronic *P. aeruginosa* lung infections (Pedersen et al., 1990). This was one of the earliest reports linking the regulation of pyoverdine production to virulence factors that are not directly related to iron homeostasis.

To gain better insight into pyoverdine regulation, we performed a high-throughput genetic screen in *P. aeruginosa* to identify genes necessary for pyoverdine production. A substantial fraction of gene hits were involved in initial attachment and biofilm formation, particularly those responsible for flagellin synthesis, chemotaxis, and type IV pili synthesis. This suggests that biofilm formation is required for pyoverdine production. We also showed that disrupting biofilm formation either genetically or chemically was sufficient to rescue *C. elegans* from pyoverdine-mediated pathogenesis. We demonstrated that cyclic-diguanylate monophosphate (c-di-GMP), a secondary messenger and master regulator of virulence factors in the pathogen, regulates pyoverdine production in a biofilm-dependent manner. On this basis, we discovered that 2-amino-5,6-dimethylbenzimidazole, a biofilm inhibitor, inhibits pyoverdine virulence.

## Materials and methods

### Strains and growth conditions

Strains used are listed in Table 1. For all pyoverdine production and biofilm formation assays, bacteria were seeded in M9 media (M9 salts (1% w/v) and casamino acids (1% w/v), supplemented with 1 mM MgSO_4_ and 1 mM CaCl_2_) in static six-well plates (Greiner, North Carolina) at 30°C. SK media was composed of 0.35% (w/v) Bacto-Peptone and 0.3% (w/v) NaCl, supplemented with 1 mM MgSO_4_ and 1 mM CaCl_2_ (Conery et al., 2014).

**Table 1:** Plasmids and strains used in this study.

**Strains**	**Relevant genotype**	**Source or reference**
**PLASMIDS**		
Constitutive RFP Expression	pUC19::*dsRED* Amp^R^	Kirienko et al., 2013
*pvdA* reporter	Mini-CTX2::P*pvdA*-*gfp* Tc^R^	Yang et al., 2009
***P. aeruginosa*** **PA14 STRAINS**		
PA14	WT	Rahme et al., 1995
Δ*pelA*	PA14*ΔpelA*	Kuchma et al., 2007
Δ*flgK*	PA14*ΔflgK*	Shanks et al., 2006; Kuchma et al., 2010
Δ*pilY1*	PA14*ΔpilY1*	Kuchma et al., 2010
Δ*motAB*	PA14*ΔmotAΔmotB*	Kuchma et al., 2015
Δ*pvdA*	PA14*ΔpvdA*	Shanks et al., 2006; Kuchma et al., 2010
*pvdE*	PA14*pvdE* Gent^R^	Liberati et al., 2006
Δ*sadC*	PA14*ΔsadC*	Merritt et al., 2007
Δ*bifA*	PA14*ΔbifA*	Kuchma et al., 2007
Δ*bifA*Δ*pelA*	PA14*ΔbifAΔpelA*	Kuchma et al., 2007
***P. aeruginosa*** **PAO1 STRAINS**		
PAO1	WT	Holloway et al., 1994
*pelB*	PAO1*pelB* Tc^R^	Jacobs et al., 2003
*fliF*	PAO1*fliF* Tc^R^	Jacobs et al., 2003
*pilG*	PAO1*pilG* Tc^R^	Jacobs et al., 2003
*motA*	PAO1*motA* Tc^R^	Jacobs et al., 2003
***C. elegans*** **STRAINS**		
Temperature sensitive mutant	*glp-4(bn2ts)*	Beanan and Strome, 1992

*Amp^R^: ampicillin resistant*.

*Tc^R^: tetracycline resistant*.

*Gent^R^: gentamicin resistant*.

### Transposon mutant library screen

PA14 transposon mutants were inoculated into 96-well plates with LB media containing 15 μg/mL gentamicin. Inoculated plates were incubated overnight at 35°C. 10 μL of LB culture from each well were transferred into 96-well, clear, flat-bottom plates (Greiner, North Carolina) containing 90 μL of M9 media per well. Bacterial plates were grown at room temperature for 24 h. Pyoverdine production (Ex 405 nm, Em 460 nm) and bacterial growth (O.D._600_) were measured every hour in these plates using a Cytation5 (BioTek, Vermont) multimode plate reader.

### Pyoverdine production kinetics assay

Bacterial strains were grown in LB media with appropriate antibiotics overnight with shaking. Two milliliters M9 media were dispensed into each well in six-well plates (Greiner, North Carolina) and inoculated with 100 μL from overnight LB cultures. The plate was incubated at 30°C inside a plate reader for 24 h with pyoverdine fluorescence measurements and bacterial growth absorbance measurements made every 30 min. Each experiment consisted of at least three biological replicates.

### Biofilm formation assay

This procedure was adapted from (Merritt et al., 2005). In brief, bacterial strains were grown under conditions identical to those described above. After incubation at 30°C for 24 h, bacterial cultures were aspirated and the biofilm matrix on the bottom of the plate was stained with 2 mL of 0.1% (w/v) crystal violet in 20% (v/v) ethanol/water for 30 min. The stain was removed and excess stain was washed with two consecutive rinses of PBS (Gibco, Maryland). Plates were dried and then photographs were taken. For biofilm quantification, the remaining crystal violet was solubilized in 30% acetic acid and absorbance was measured at 550 nm. Each experiment consisted of at least three biological replicates. Statistical significance was determined using Student's *t*-test.

### RNA purification and qRT-PCR

After 8 h growth in six-well plates, planktonic cells were collected from 1.5 mL of supernatant. RNA was extracted and purified using Trizol reagent (Invitrogen, California) according to manufacturer's protocols with minor adjustments. To ensure cell lysis, cells resuspended in Trizol reagent were heated at 95°C for 15 min prior to phase separation. Purified RNA was treated with DNase I (Thermo Scientific, Massachusetts). Reverse transcription was performed using random decamers and Retroscript kit (Ambion). qRT-PCR was conducted using SYBR green PerfeCTa SYBR Green Fastmix (Quantabio, Massachusetts) in a CFX-96 real-time thermocycler (Bio-Rad, California). Fold-changes were calculated using a ΔΔCt method, and compared to expression from wild-type *P. aeruginosa*. Primer sequences are available upon request.

### Biofilm and planktonic cells pyoverdine expression measurement

Two milliliters of M9 media were dispensed into three wells of a six-well plate (Greiner, North Carolina) and inoculated with 100 μL of PA14 *dsRed*/P*pvdA*-*gfp* LB overnight culture. After incubation at 30°C for 16 h, supernatant was carefully collected from each well. Planktonic cells were collected from 1 mL of media. Cells in biofilm were collected by scraping all three wells into 1 mL of PBS buffer using a cell scraper. The two cells samples were extensively washed and resuspended in 1 mL of PBS. GFP and dsRed fluorescence from 100 μL of each sample was measured in a black 96-well plate (Greiner, North Carolina). Each experiment consisted of at least three biological replicates. Statistical significance was determined using Student's *t*-test.

### Biofilm and planktonic cells pyoverdine imaging

Two milliliters of M9 media were dispensed into each of three wells in a six-well plate (Greiner, North Carolina) and inoculated with 100 μL of *P. aeruginosa* grown overnight in LB. After 8 or 16 h incubation at 30°C, supernatant was carefully collected from all wells. Planktonic cells were collected from 2 mL of media. Planktonic cells and biofilm-associated cells attached to the bottom of the plate were washed twice with PBS. Planktonic cells were resuspended in 0.5 mL PBS buffer and dispensed into a six-well plate. Biofilms and concentrated planktonic cells were imaged using a custom filter (445/45 excitation, 510/42 emission, 482 nm dichroic) using a Cytation5 multimode reader (Biotek, Vermont). All images were taken under identical conditions, and each experiment consisted of at least three biological replicates.

### *C. elegans* pathogenesis assay

The procedure was adapted from (Kirienko et al., 2014). In brief, *glp-4*(*bn2ts*) worms were grown at 25°C on *E. coli* OP50 until they reached young adulthood. Worms were washed off plates, rinsed with S Basal, and resuspended in M9 media (500 worms/mL). 1,000 worms were dispensed into each well of a six-well plate and inoculated with 100 μL of overnight *P. aeruginosa* PA14 culture grown in LB. Plates were covered with air-permeable membranes and incubated at 25°C for 30 h. After exposure to *P. aeruginosa*, worms were collected from the wells and extensively washed in S Basal buffer. Worms were pelleted by gravity between washes to effectively remove most *P. aeruginosa* bacteria from the media. After washes, 30–40 worms were dispensed into each well of black half-area 96-well clear bottom plates (Greiner, North Carolina). Worms were stained with Sytox Orange nucleic acid stain (Thermo Fisher) for 12 h at room temperature. Excess stain was removed by washing twice in S Basal, and plates were imaged under bright field and RFP channels using a Cytation5 multimode plate reader (BioTek, Vermont). Relative host death was quantified by normalizing RFP/bright field intensity in worms. Killing of *C. elegans* by biofilm mutants was normalized to that of wild-type *P. aeruginosa*. Each experiment consisted of at least three biological replicates. Each biological replicate contained 12 wells (~400 worms). Statistical significance was determined using Student's *t*-test.

### *C. elegans*' ferric iron quantification

*glp-4*(*bn2ts*) worms were grown at 25°C on *E. coli* OP50 until they reached young adulthood. Worms were washed off plates, rinsed with S Basal, and resuspended in M9 media (3,000 worms/mL). 6,000 worms were dispensed into each well of a six-well plate and inoculated with 100 μL of *P. aeruginosa* PA14 grown overnight in LB. Three wells were used for each condition for each biological replicate. Plates were covered with air-permeable membranes and incubated at 25°C for 36 h. Worms were collected and extensively washed in S Basal buffer. To facilitate lysis, worms were frozen at –80°C, thawed, and sonicated. To quantify iron (III) concentration, 50 μL of pyoverdine-rich bacterial filtrate of known fluorescence was added to 200 μL of lysate. After incubation, pyoverdine fluorescence was measured with a Cytation5 (Biotek, Vermont), and was compared to the same filtrate diluted with media. Since pyoverdine binds to ferric iron in a 1:1 stoichiometric ratio, the reduction of pyoverdine fluorescence reflects the ferric iron content in the worm lysate. Iron concentrations in worms exposed to biofilm mutants and to wild-type *P. aeruginosa* PA14 were compared. Each experiment consisted of at least three biological replicates. Statistical significance was determined using Student's *t*-test.

### Quantification of biofilm and pyoverdine in different surface growth conditions

Twenty-five microliters of *P. aeruginiosa* PA14 grown overnight in LB was inoculated into 475 μL of M9 media in 17 mm diameter glass or plastic culture tubes. Tubes were grown in 30°C under static growth conditions for 16 h. Bacterial supernatant was collected from the tubes, and pyoverdine fluorescence was determined. Biofilms in tubes were stained and crystal violet staining was measured as described above. Each experiment consisted of at least three biological replicates. Statistical significance was determined using Student's *t*-test.

## Results

### Transposon mutant library screen reveals a link between biofilm formation and pyoverdine production

**Figure 1:**
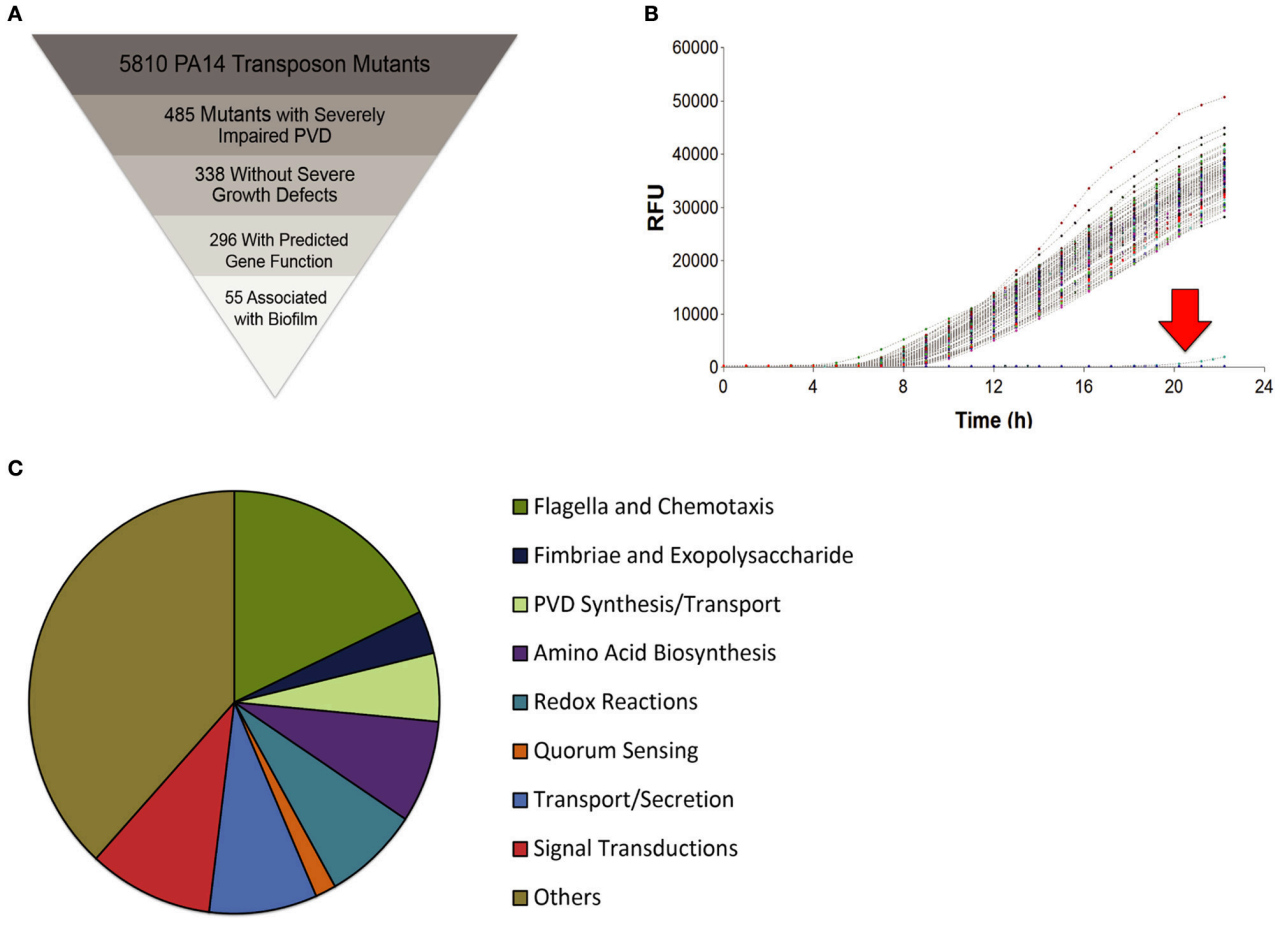
PA14 transposon mutant library screen for genes essential for pyoverdine production. **(A)** Schematic diagram of library screening process with criteria for identifying screen hits. **(B)** Kinetic data from a sample plate showing two mutants with severely impaired pyoverdine production (< 30% of normal, below the red arrow). **(C)** Families of gene functions identified and their relative enrichment among hits identified in the screen.

Using a non-redundant *P. aeruginosa* PA14 transposon mutant library (Liberati et al., 2006), we conducted a high-throughput, kinetic screen of 5,810 mutants to measure pyoverdine production. The high-throughput screen was performed under static growth conditions in 96-well plates, which resulted in robust biofilm formation in most wells, supporting the potential for a relationship between the generation of biofilm and production of pyoverdine. Fluorescence spectrophotometry (Ex 405 nm, Em 460 nm) was used to monitor pyoverdine biosynthesis in 96-well plates over a 24 h span. Of the mutants screened, 485 showed severe impairments in pyoverdine fluorescence (as defined as production below 30% of wild-type levels). Eliminating targets with growth defects (i.e., mutants where reduced pyoverdine biosynthesis might be caused by poor growth) left 338 hits. These were narrowed further to include only 296 hits that had predicted gene function (Figures 1A,B). 55 of these (18.6%) were associated with various stages of biofilm formation (Figure 1C), including flagellin biosynthesis, chemotaxis, type IV pili assembly, Cup fimbriae biogenesis, and exopolysaccharide synthesis (Table S1).

**Figure 2:**
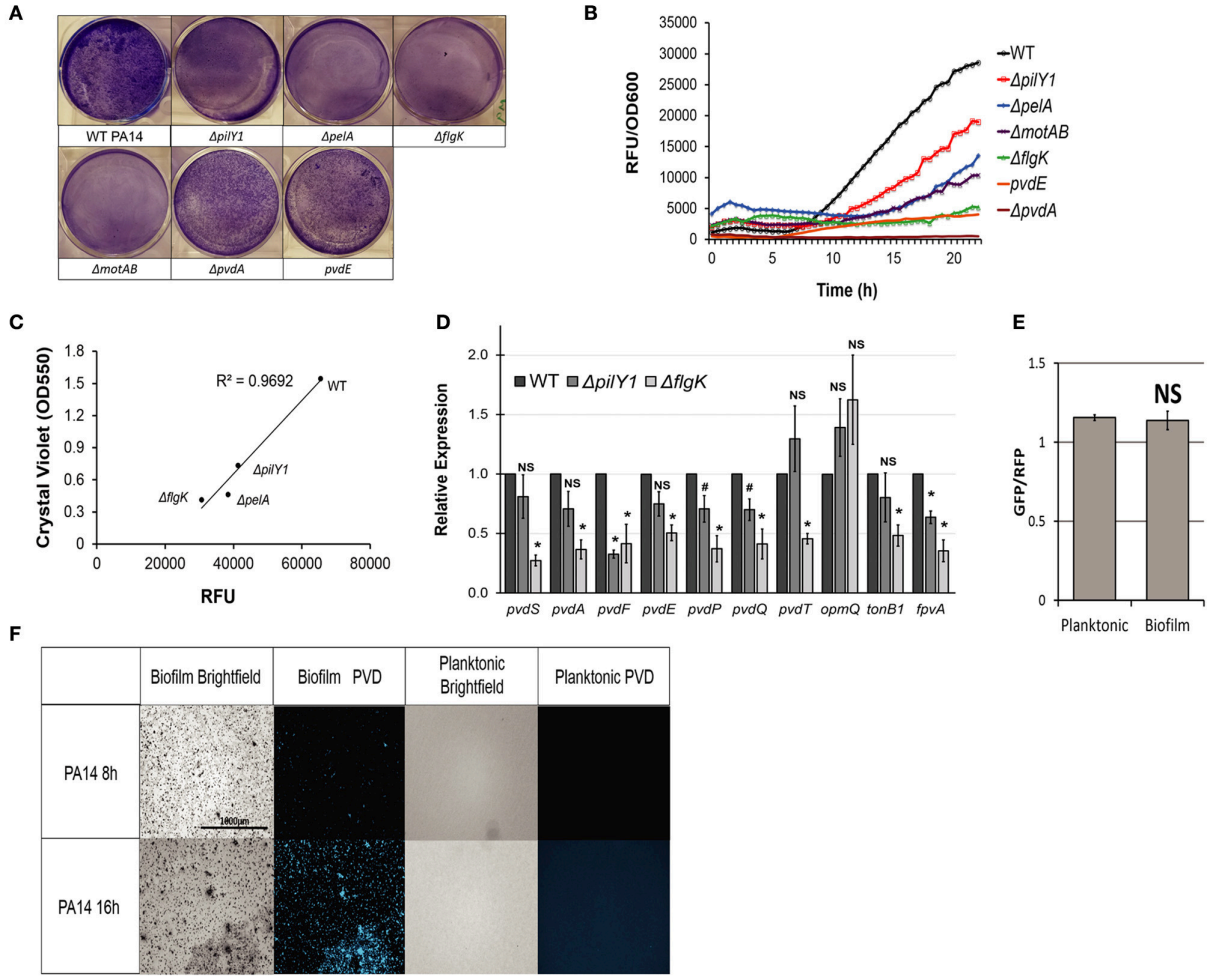
Biofilm formation is necessary for pyoverdine production. **(A)** Biofilm matrix of PA14 or 6 PA14 mutants in six-well plates stained with 0.1% crystal violet solution. **(B)** Pyoverdine fluorescence normalized to bacterial growth, measured kinetically over 24 h in biofilm mutants. **(C)** Scatterplot of pyoverdine and biofilm produced in PA14 biofilm mutants grown in 6-well plate cultures. Biofilm was quantified by resuspending crystal violet stain in 30% acetic acid solution and measuring absorbance at 550 nm. **(D)** qRT-PCR of genes involved in pyoverdine biosynthesis, secretion, and signaling in WT *P. aeruginosa* PA14, Δ*pilY1* mutant, and Δ*flgK* mutant. Gene expressions in biofilm mutants are normalized to those in wild-type. **(E)** Pyoverdine expression in planktonic and biofilm cells after 16 h growth measured by GFP fluorescence from P*pvdA-gfp* construct normalized to constitutively expressed dsRed fluorescence. **(F)** Pyoverdine production in PA14 biofilm matrix and planktonic cells imaged using pyoverdine-specific fluorescence filter. Data presented in **(A–C)**, and **(F)** are representative results from three biological replicates. Error bars in **(D,E)** represent SEM between three biological replicates. NS corresponds to *p* > 0.05, # corresponds to *p* < 0.05, and ^*^ corresponds to *p* < 0.01 (based on Student's *t*-test). Pyoverdine production curves without bacterial growth normalization are available in Figure S6.

To verify our findings, we selected genes representative for each stage of biofilm formation (initial surface attachment: *flgK*, a flagellum biosynthesis gene, and *motAB*, required for chemotaxis; adhesion factor production: *pilY1*, a type IV pili mutant; and bioflm maturation: *pelA*, an exopolysacharide mutant) (Vogeleer et al., 2014). As expected, these strains exhibited impairments in both biofilm formation (Figure 2A) and pyoverdine production (Figure 2B). We also observed a strong linear correlation between the amount of biofilm and pyoverdine produced by these mutants (Figure 2C), indicating a role for biofilm formation in pyoverdine production.

Quantitative real-time PCR (qRT-PCR) using RNA harvested from WT *P. aeruginosa* PA14, Δ*pilY1*, and Δ*flgK* planktonic cells after 8 h growth corroborated this finding. We used qRT-PCR to analyze expression of the alternative sigma factor *pvdS* and *pvdS*-dependent pyoverdine biosynthesis genes [including *pvdA* and *pvdF* (which are responsible for generating the non-standard amino acid *N*-formyl-*N*-hydroxyornithine), *pvdE* (predicted to transport nascent pyoverdine into the periplasm), *pvdP* and *pvdQ* (involved in periplasmic maturation)] (Visca et al., 2007; Drake and Gulick, 2011). All genes were significantly downregulated in Δ*flgK* mutant (Figure 2D). As may be hypothesized by the weaker pyoverdine biosynthesis phenotype (Figure 2B), the Δ*pilY1* mutant showed significantly less impact, although several genes were still disrupted, including *pvdF, pvdP*, and *pvdQ*. The PvdRT-OpmQ system is thought to be responsible for recycling pyoverdine back out of the periplasmic space (Imperi et al., 2009). Unsurprisingly (*pvdT* is also transcriptionally regulated by *pvdS*), *pvdT* expression was diminished in the Δ*flgK* mutant, although regulation of *opmQ*, which is in the same operon as *pvdR* and *pvdT*) was unaffected. Our qRT-PCR data also suggest a possible role for ferripyoverdine uptake in biofilm-mediated regulation of pyoverdine production. *fpvA*, the ferripyoverdine receptor (Shen et al., 2002), and *tonB1*, which provides the energy for ferripyoverdine translocation into the cell (Shirley and Lamont, 2009), were significantly downregulated in the Δ*flgK* mutant (Figure 2D).

To determine whether the regulatory relationship between pyoverdine and biofilm is bidirectional (i.e., whether pyoverdine production was necessary for biofilm formation), we assayed biofilm development in two mutants (PA14Δ*pvdA* and PA14*pvdE*). Pyoverdine production was abolished in PA14Δ*pvdA* (Figure 2B), while PA14*pvdE* produced a small amount of pyoverdine (Figure 2B). Both mutants showed nearly normal biofilm formation (Figure 2A). This is consistent with previous results from Banin and colleagues, who showed that active iron acquisition was necessary for biofilm formation, but that pyoverdine itself is not (Banin et al., 2005).

*P. aeruginosa* strains exhibit tremendous variation in the regulation of virulence genes (Lee et al., 2006). Therefore, we tested whether biofilm formation was necessary for pyoverdine production in PAO1, the reference strain for *P. aeruginosa*. PAO1 mutants with transposons inserted into analogous genes involved in biofilm formation (*fliF* and *motA* to disrupt attachment, *pilG* to disrupt adhesion, and *pelB* to disrupt biofilm maturation) generally exhibited compromised pyoverdine production (Figures S1A,B). However, unlike PA14Δ*pelA* mutant, the PAO1*pelB* strain was capable of forming dense biofilms comparable to wild-type bacteria (Figure S1A), resulting in wild-type levels of pyoverdine production (Figure S1B). This is likely due to the fact that PAO1 produces two different types of exopolysaccharides, Pel and Psl, and the disruption in Pel alone is insufficient to effectively decrease biofilm formation and pyoverdine biosynthesis. This is consistent with our observations that PAO1 appears to create more biofilm than PA14 (Figure S2A). Interestingly, PAO1 also secretes more pyoverdine and at a faster rate than PA14 (Figure S2B). However, in genes that most disrupt biofilm formation (*flgK* for PA14 and *motAB* for PAO1), pyoverdine production was attenuated to similar levels in the two strains (Figure 2B, Figure S1B).

Based on these observations, we hypothesized that sessile and planktonic cells would exhibit differences in pyoverdine biosynthetic machinery. We used a GFP-based reporter to assay expression of the *pvdA* gene, which was normalized to constitutively-expressed dsRed. To our surprise, *pvdA* transcription was indistinguishable between planktonic and biofilm-associated cell populations (Figure 2E). However, we observed significant differences in pyoverdine production between sessile and planktonic cells in earlier stages of bacterial growth when pyoverdine biosynthesis is initiated. Using fluorescence microscopy, we visualized intracellular pyoverdine levels in sessile cells aggregated in the biofilm matrix and planktonic cells collected from the growth media after 8 h (when pyoverdine production is initiated), and 16 h of growth (Figure 2F). At 8 h, pyoverdine fluorescence is detected only in cells in the biofilm matrix, but at 16 h, pyoverdine fluorescence is present in both biofilm matrix and concentrated planktonic cells (Figure 2F). In a Δ*pvdA* mutant, neither biofilm matrix cells nor planktonic cells exhibit fluorescence, verifying that the fluorescence detected is from pyoverdine (Figure S3A). These results suggest one possible model for biofilm-dependent regulation of pyoverdine. First, pyoverdine production is initiated in the microcolonies that will nucleate biofilm formation. Secreted pyoverdine binds to ferric iron, returning to the pathogen via the ferripyoverdine receptor protein FpvA (Shen et al., 2002). This increases the activity of PvdS, upregulating pyoverdine biosynthesis genes in both sessile and planktonic cells (Lamont et al., 2002; Llamas et al., 2014). Attenuated pyoverdine fluorescence in the extracellular matrices of biofilm mutants (Figure S3B) and the downregulation of ferripyoverdine uptake genes in Δ*pilY1* and Δ*flgK* planktonic cells (Figure 2D) are consistent with this model.

### Disrupting biofilm formation alleviates pyoverdine-mediated virulence in *C. elegans*

**Figure 3:**
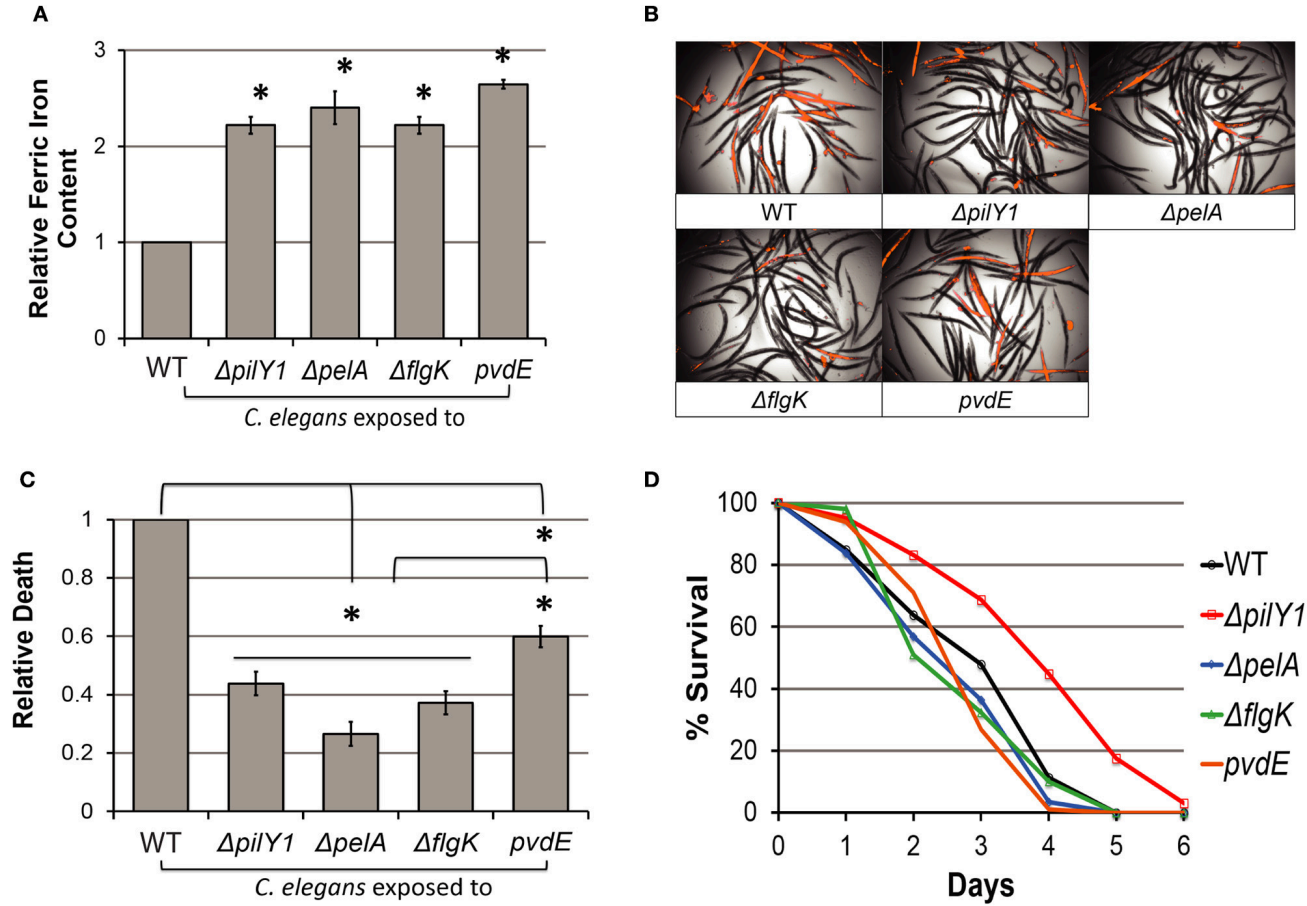
Impairment of biofilm formation mitigates pathogen virulence. **(A)** Relative ferric iron content in *C. elegans* lysates from worms exposed to PA14 biofilm mutants. Detailed procedure is described in Materials and Methods. **(B)** Sytox Orange stain in worms exposed to PA14 biofilm mutants visualized by RFP channel image merged with brightfield. **(C)** Host death among PA14 mutants was quantitatively compared by RFP fluorescence normalized to brightfield intensity. Relative host death in biofilm mutants was normalized to death observed in worms exposed to wild-type PA14. **(D)** *C. elegans* slow kill assay survival curve for PA14 biofilm mutants. Data presented in **(B–D)** are representative results from three biological replicates. Error bars in **(A)** represent SEM between three biological replicates. Error bars in **(C)** represent SEM between 12 technical replicates. Asterisks indicate significant difference between conditions (*p*-value < 0.01, based on Student's *t*-test).

Pyoverdine is mainly involved in iron transport, and its exceptionally high affinity for ferric iron grants the pathogen a significant advantage over the host in the competition for iron acquisition. We recently reported that exposure to pyoverdine significantly disrupts host iron homeostasis, inducing a lethal hypoxic response in *C. elegans* (Kirienko et al., 2013). Based on these findings, we predicted that the attenuation of pyoverdine production in *P. aeruginosa* biofilm mutants would limit their virulence and their ability to remove iron from the host. We used a fluorometric assay to determine relative ferric iron concentration in *C. elegans* (see *Materials and Methods* for details). As expected, *C. elegans* exposed to *pelA, flgK*, or *pilY1* mutants exhibited significantly greater ferric iron retention than pyoverdine-competent controls, similar to worms exposed to PA14*pvdE* (Figure 3A).

To determine whether this was sufficient to alleviate pyoverdine-mediated pathology, we exposed worms to wild-type or biofilm mutants of *P. aeruginosa* for 30 h, washed them extensively to remove the pathogen, and then stained them with Sytox Orange, a cell-impermeant dye that stains only dead worms. *C. elegans* exposed to biofilm mutants displayed significant reductions in mortality compared to wild-type *P. aeruginosa* (Figures 3B,C). Interestingly, biofilm mutations compromised virulence to a greater extent even than the *pvdE* mutant (Figures 3B,C). This suggests that biofilm formation may contribute to virulence through multiple means.

To identify the effects of biofilm formation in a pathogenesis model that is not dependent upon pyoverdine, we tested whether biofilm mutants showed attenuation in an agar-based, intestinal colonization assay known as Slow Killing (Kirienko et al., 2013). With the exception of PA14 Δ*pilY1*, the biofilm mutants showed wild-type levels of virulence (Figure 3D). These data were also corroborated by screening results from Feinbaum and colleagues (Feinbaum et al., 2012). A majority of biofilm mutants (again, with the exception of mutants that compromised formation of the type IV pili) had no effect in *P. aeruginosa*'s ability to kill *C. elegans*.

### Intracellular c-di-GMP levels modulate pyoverdine production by regulating biofilm

**Figure 4:**
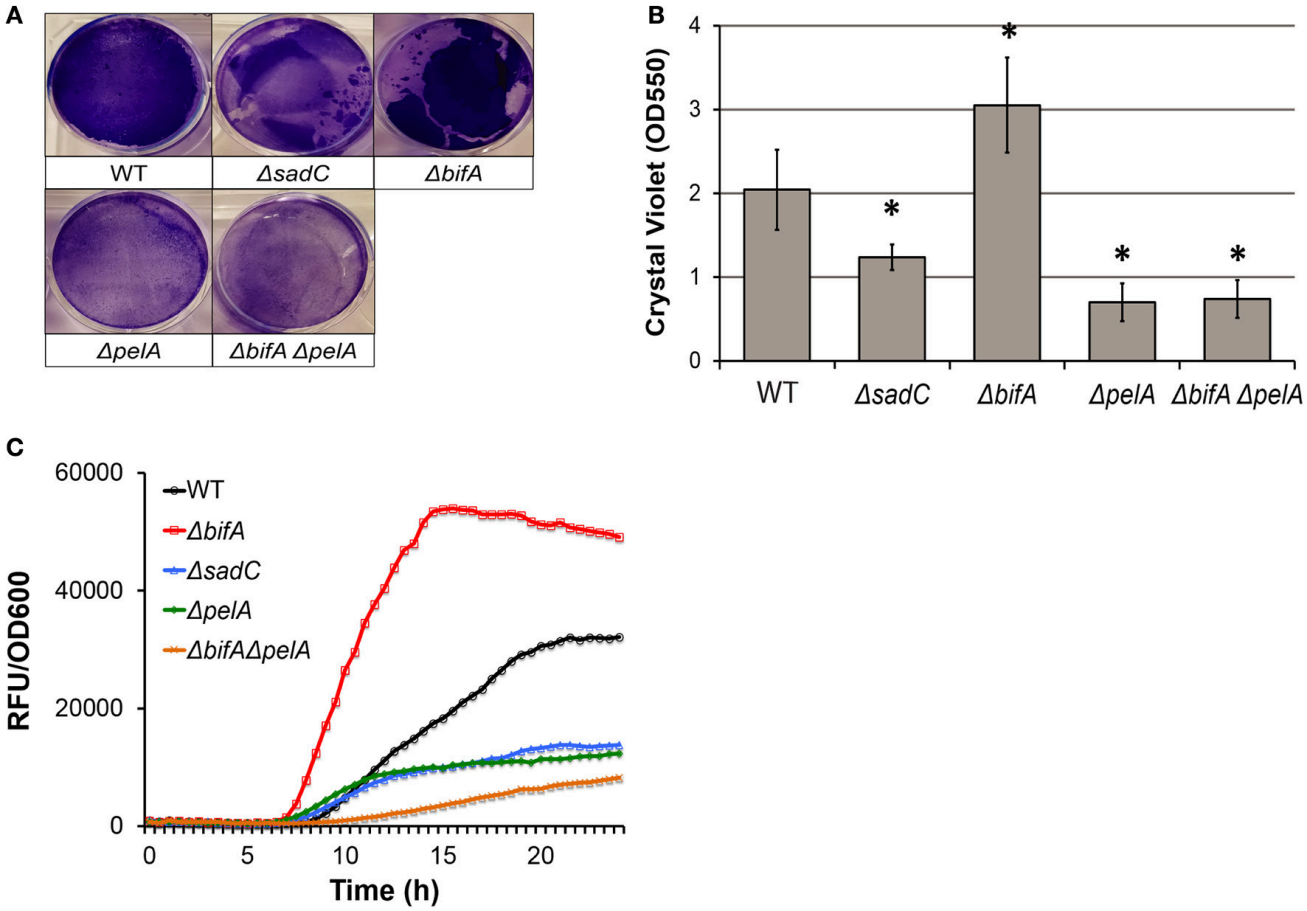
Intracellular c-di-GMP concentrations modulate pyoverdine production in a biofilm-dependent manner. **(A)** Biofilm matrix of c-di-GMP biosynthesis mutants stained with 0.1% crystal violet solution. **(B)** Crystal violet concentration measured by absorbance at 550 nm after biofilm matrix stain was solubilized in 30% acetic acid. **(C)** Pyoverdine fluorescence normalized to bacterial growth was measured kinetically over 24 h in diguanylate cyclase and phosphodiesterase mutants and the phosphodiesterase mutant with biofilm defect. Data presented in **(A,C)** are representative results from three biological replicates. Error bars in **(B)** represent SEM between three biological replicates. Asterisks indicate significant difference between conditions (*p*-value < 0.01, based on Student's *t*-test). Pyoverdine production curves without bacterial growth normalization are available in Figure S6.

Cyclic diguanylate monophosphate (c-di-GMP) is a crucial secondary messenger in *P. aeruginosa* that transcriptionally regulates a wide variety of virulence factors, including type III and type VI secretion (Moscoso et al., 2011). Previous research has also linked intracellular c-di-GMP concentration to biofilm formation. For example, diguanylate cyclases (i.e., SadC) are involved in c-di-GMP synthesis and support increased formation of biofilms (Kuchma et al., 2007; Merritt et al., 2007), while phosphodiesterases (i.e., BifA) hydrolyze c-di-GMP and limit biofilm formation (Kuchma et al., 2007; Merritt et al., 2007). Under our screening conditions, PA14Δ*sadC* (low c-di-GMP) and PA14Δ*bifA* (high c-di-GMP) mutants exhibited less and more biofilm respectively (Figures 4A,B), which is consistent with previously published data (Kuchma et al., 2007; Merritt et al., 2007). As predicted, pyoverdine production was attenuated in Δ*sadC* and enhanced in Δ*bifA* mutants (Figure 4C).

To test the regulatory relationship between c-di-GMP concentration and biofilm formation in pyoverdine production, we repeated the kinetics experiment with a PA14Δ*bifA*Δ*pelA* double mutant that exhibits high intracellular c-di-GMP concentrations (Kuchma et al., 2007), but poor biofilm formation (Figures 4A,B). Since pyoverdine production was not completely abolished in the PA14Δ*pelA* mutant background, the ramifications of *bifA* deletion on pyoverdine production (whether augmented or decreased) can be observed. Pyoverdine production in the PA14Δ*bifA*Δ*pelA* double mutant was similar, if not lower, to Δ*pelA* single mutant (Figure 4C), indicating that regulation of pyoverdine via intracellular c-di-GMP concentrations is hypostatic to biofilm formation. This confirms that c-di-GMP's effect on pyoverdine production is mediated indirectly, via biofilm formation.

### Chemical and physical modifiers of biofilm formation affect pyoverdine production

**Figure 5:**
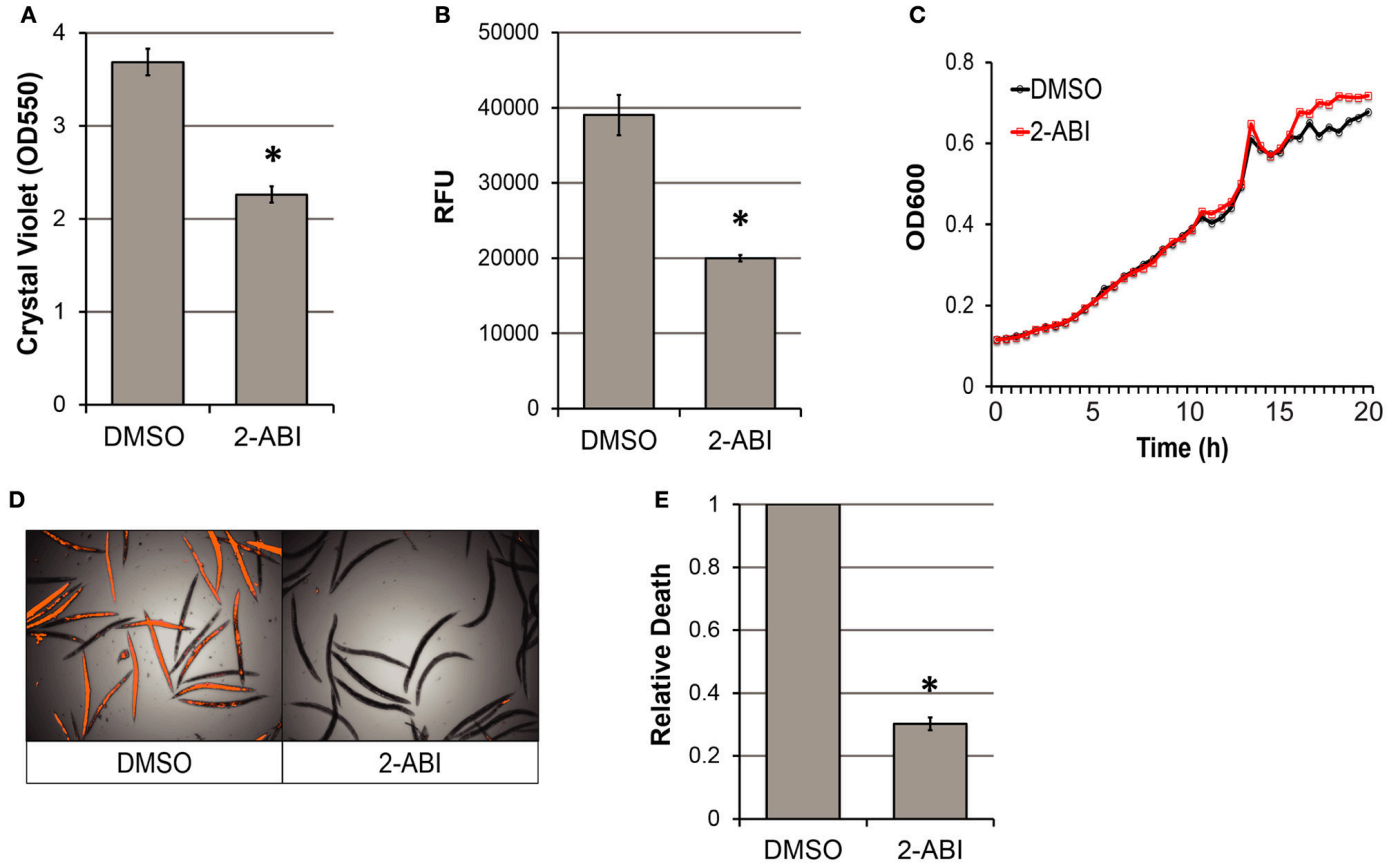
Biofilm inhibitor significantly impairs pyoverdine production and attenuate *P. aeruginosa* virulence. **(A)** Quantification of crystal violet-stained biofilm matrix solubilized in acetic acid for PA14 grown in M9 media with 25 μM 2-ABI or DMSO solvent control. **(B)** Pyoverdine fluorescence of bacterial supernatant after 24 h of static growth for PA14 grown in conditions as above. **(C)** Bacterial growth measured kinetically over 24 h by absorbance at 600 nm for PA14 grown in conditions as above. **(D)** Sytox Orange stain in worms exposed to PA14 in the presence of DMSO solvent or 25 μM 2-ABI visualized by RFP channel image merged with brightfield image. **(E)** Host death under the two conditions was quantitatively compared by RFP fluorescence normalized to brightfield intensity. Host death in the presence of 2-ABI was normalized to that of DMSO control. All data presented are representative results from biological replicates. Error bars in **(A,B,E)** represent SEM between 12 technical replicates. Asterisks indicate significant difference between conditions (*p*-value < 0.01, based on Student's *t*-test).

We hypothesized that chemical inhibition of biofilm formation, like genetic disruption, would reduce pyoverdine production. Several compounds are known to compromise *P. aeruginosa* biofilm formation, including the aromatic heterocycle 2-amino-5,6-dimethylbenzimidazole (2-ABI) (Frei et al., 2012). Addition of 25 μM 2-ABI to *P. aeruginosa* culture reduced biofilm formation by 40% compared to the solvent control (Figure 5A), with a concomitant decrease (60%) in pyoverdine production (Figure 5B). The decrease in these exoproducts was not a consequence of bacterial growth inhibition; bacterial titer was unaffected by the presence of the compound (Figure 5C). As expected, disruption of biofilm formation and pyoverdine production by the addition of 2-ABI were sufficient to rescue *C. elegans* (Figures 5D,E), offering an exciting possibility of utilizing this molecule as an anti-virulent.

Another way to significantly alter biofilm formation is by changing the surface characteristics of the vessel for *P. aeruginosa* static cultures. In this case many of the off target effects that may be induced by chemical inhibitors or genetic manipulations will be eliminated. On a plastic surface, *P. aeruginosa* PA14 formed approximately one-half of the biofilm as a glass surface (Figure S4A). This led to ~25% decrease in pyoverdine production (Figure S4B).

### Pyoverdine production is also regulated by iron starvation and nutrient limitation

**Figure 6:**
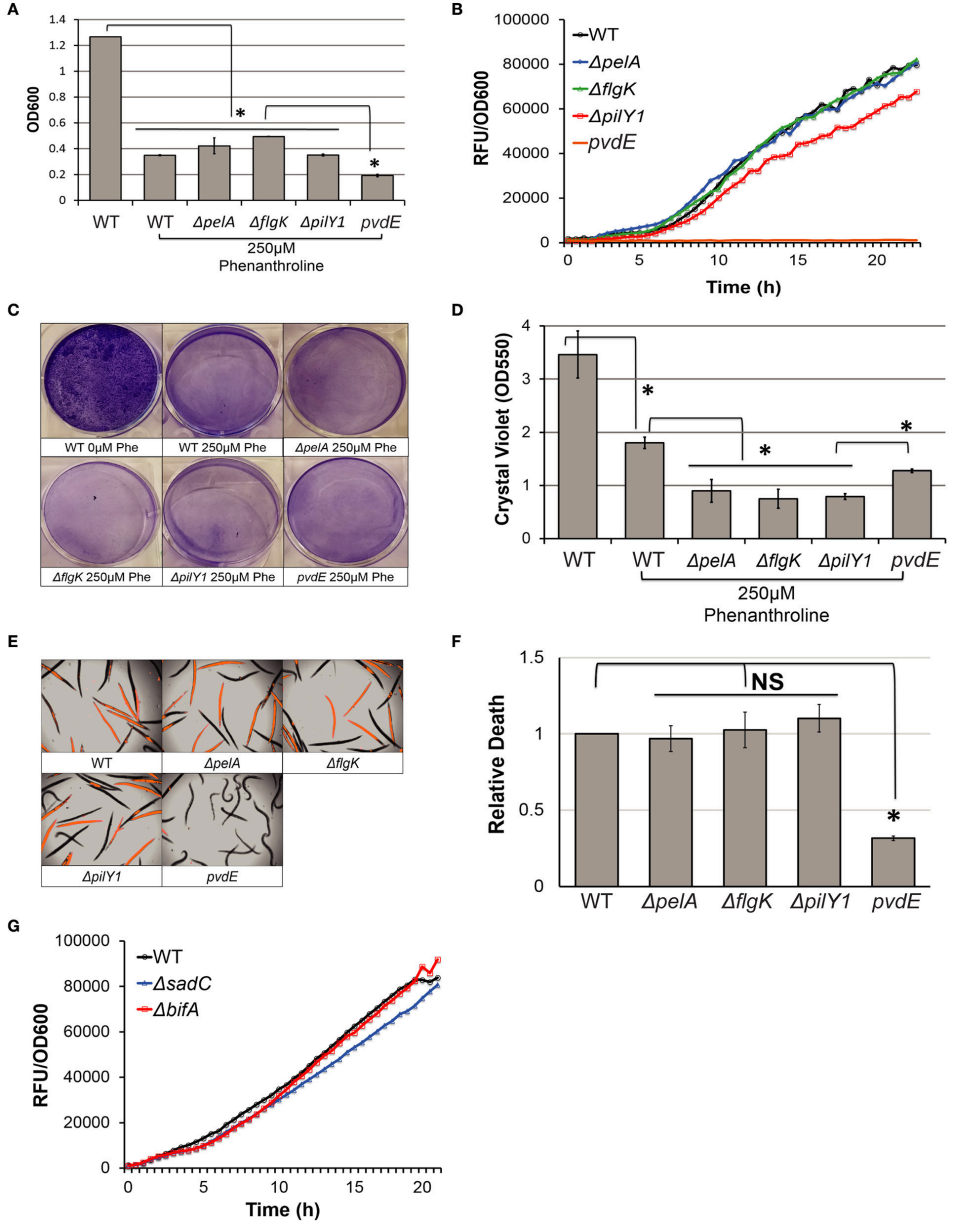
Biofilm formation doesn't affect pyoverdine production under iron-starved conditions. **(A)** Bacterial growth for PA14 biofilm mutants grown in M9 media with 250 μM 1,10-phenanthroline measured by absorbance at 600 nm after 24 h incubation at 30°C. **(B)** Pyoverdine fluorescence normalized to bacterial growth kinetically measured over 24 h in biofilm mutants grown in the presence of 250 μM phenanthroline. **(C)** Crystal violet-stained biofilm matrices of biofilm mutants grown in the presence of phenanthroline. **(D)** Quantification of crystal violet stain by solubilizing stain in 30% acetic acid and measuring absorbance at 550 nm. **(E)** SYTOX Orange stain in worms exposed to PA14 biofilm mutants grown in the presence of phenanthroline visualized by RFP channel image merged with brightfield. **(F)** Host death among PA14 mutants was quantitatively compared by RFP fluorescence normalized to brightfield intensity. Relative host death in biofilm mutants was normalized to death observed in worms exposed to wild-type PA14. **(G)** Pyoverdine fluorescence normalized to bacterial growth kinetically measured over 24 h in c-di-GMP biosynthesis mutants grown in the presence of 250 μM phenanthroline. Data are representative results from three biological replicates. Error bars in **(A,D,F)** represent SEM between 12 technical replicates. Asterisks indicate significant difference between conditions (*p*-value < 0.01, based on Student's *t*-test). NS corresponds to *p* > 0.05. Pyoverdine production curves without bacterial growth normalization are available in Figure S6.

Arguably, the most important cellular function for pyoverdine is iron acquisition. Since biofilm formation is necessary for pyoverdine production, we hypothesized that biofilm mutants would be compromised under conditions where pyoverdine is required for growth. To test this hypothesis, we assessed their growth alongside PA14*pvdE* mutant in the presence of 1,10-phenanthroline, a ferrous iron chelator (Figure 6A). When grown in media supplemented to a final concentration of 250 μM 1,10-phenanthroline, growth was compromised in all strains, but it was most severely curtailed in *pvdE* mutant. To our surprise, neither growth nor pyoverdine production in biofilm mutants in low iron conditions were significantly different from wild-type bacteria (Figures 6A,B). This was not due to improvement of biofilm formation; mutants still showed significantly reduced biofilm under these conditions (Figures 6C,D). This observation is consistent with findings by Hunter et al., who reported that inhibiting ferrous iron acquisition in *P. aeruginosa* reduces biofilm formation (Hunter et al., 2013). The recovery of pyoverdine production in biofilm mutants treated with 1,10-phenanthroline demonstrates that biofilm formation and pyoverdine biosynthesis can be unlinked. As predicted, 1,10-phenanthroline also abolished the protection provided by biofilm gene mutation in the Liquid Killing assay (Figures 6E,F). In contrast, virulence in the *pvdE* mutant remained attenuated, even in the presence of 1,10-phenanthroline. This demonstrated that adding 1,10-phenanthroline does not abolish the importance of pyoverdine in virulence.

We observed similar results for biofilm mutants grown in the presence of the ferric iron chelator ethylenediamine-N,N′-bis(2-hydroxyphenylacetic acid (EDDHA). Addition of 1 mg/mL EDDHA significantly reduced bacterial growth and biofilm formation (Figures S5A,C). As a result, neither growth nor pyoverdine production was attenuated in biofilm mutants, but it was severely curtailed in the *pvdE* mutant (Figures S5A,B). Observations from biofilm mutants treated with 1,10-phenanthroline or EDDHA suggest that under normal conditions, biofilm production is necessary for robust pyoverdine biosynthesis, but when iron concentrations become strongly limited, pyoverdine production becomes independent of biofilm genes.

**Figure 7:**
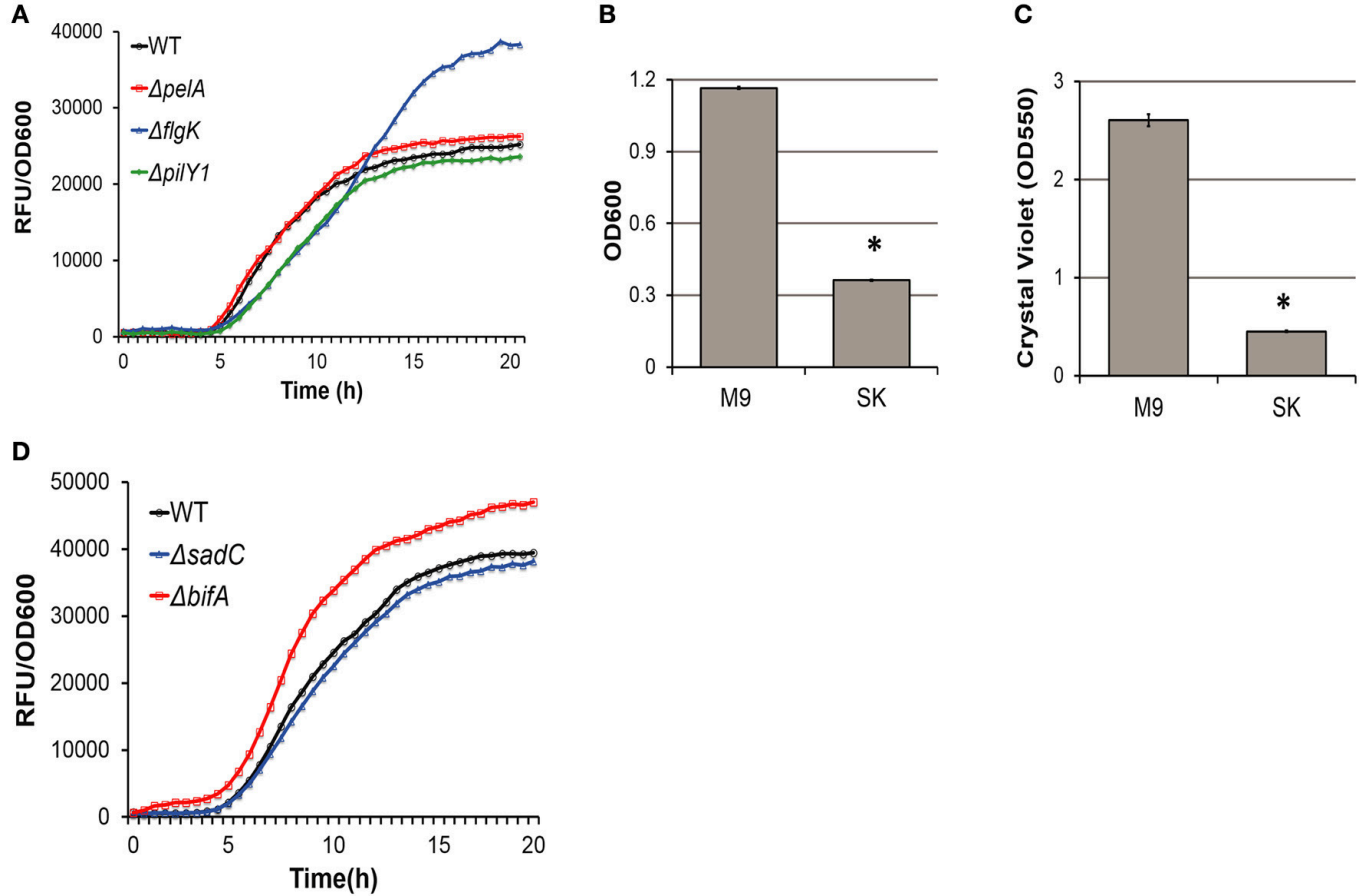
Biofilm formation doesn't affect pyoverdine production in SK media. **(A)** Pyoverdine fluorescence normalized to bacterial growth measured kinetically over 24 h in biofilm mutants grown in SK media. **(B)** Difference in bacterial growth for bacteria grown in M9 or SK media measured by absorbance at 600 nm. **(C)** Difference in biofilm formation for bacteria grown in M9 or SK media measured by crystal violet biofilm matrix stain solubilized in acetic acid. **(D)** Pyoverdine fluorescence normalized to bacterial growth measured kinetically over 24 h in c-di-GMP biosynthesis mutants grown in SK media. All error bars represent SEM between four technical replicates. Asterisks indicate significant difference between conditions (*p*-value < 0.01, based on Student's *t*-test). Pyoverdine production curves without bacterial growth normalization are available in Figure S6.

In addition to iron starvation, nutrient availability also contributes to pyoverdine regulation. Previously, we reported that biofilm mutants showed no attenuation of virulence in *C. elegans* Liquid Killing (Kirienko et al., 2013). The media used for those studies is comprised of low concentrations of sodium chloride and peptone, diluted into buffered inorganic salts to match the osmolarity of the host. This contrasts with the M9 media used in this study, which is better defined and more nutritionally rich. Kinetic measurements of biofilm mutants grown in SK media showed no significant decrease in pyoverdine fluorescence (Figure 7A). In contrast to the M9 media used in this study, SK media does not support dense bacterial growth nor formation of dense biofilms (Figures 7B,C). Much like low-iron conditions, this alleviates pyoverdine biosynthesis from biofilm regulation.

Likewise, pyoverdine production in c-di-GMP biosynthesis mutants (PA14Δ*sadC*, PA14Δ*bifA*) in the presence of 1,10-phenanthroline (Figure 6G) or SK media (Figure 7D) also did not significantly differ from that of wild-type bacteria. This further supports our model where c-di-GMP affects pyoverdine production indirectly via biofilm formation.

## Discussion

The increasing prevalence of multidrug resistant pathogens demands a new therapeutic approach to treating nosocomial infections. One possibility is to supplement antibiotics with novel drugs that compromise pathogen virulence. However, in order to develop these treatments, we need to first identify the relevant determinants and clearly understand their regulatory relationships. Toward this end, we screened a *P. aeruginosa* transposon mutant library to identify genes necessary for the production of pyoverdine. The innate fluorescence of pyoverdine enabled us to take a high-throughput, kinetic approach, monitoring pyoverdine production over 24 h. This revealed a relationship between pyoverdine, c-di-GMP, and biofilm in *P. aeruginosa*. Due to their physiological significance, these three virulence factors have been extensively studied. For example, multiple studies have demonstrated the importance of pyoverdine in various mammalian and non-mammalian models, most notably in GI tract colonization and lung infection models in mice (Imperi et al., 2013; Kirienko et al., 2013; Lopez-Medina et al., 2015; Minandri et al., 2016). The secondary messenger c-di-GMP functions as a master switch between motility and biofilm formation. Biofilms form a critical *in vivo* reservoir of infection that is particularly resistant to the immune system and antimicrobials (Costerton et al., 1999). Many previous studies have demonstrated that siderophores, including pyoverdine, play an important role in biofilm formation through their iron-scavenging activity (Banin et al., 2005; Ojha and Hatfull, 2007; Chhibber et al., 2013). Recent studies have suggested that biofilms may also regulate pyoverdine (Visaggio et al., 2015). For example, Chen et al., suggested that exopolysaccharides may regulate pyoverdine production through the Gac/Rsm pathway and the diguanylate cyclase SadC, via an unknown mechanism (Chen et al., 2015). It is difficult to know how relevant their conditions were, however, since they overexpressed the diguanylate cyclase YedQ, leading to levels of c-di-GMP that may not reflect biologically relevant conditions. Visaggio, et al., also saw a link between the Pel and Psl exopolysaccharides in pyoverdine production. However, their data suggest that the relevant function of these sugars is to drive cell aggregation in the PAO1 strain. This aggregation appears to be the driving force for pyoverdine production in their conditions (Visaggio et al., 2015).

Although our high-throughput genetic screen provided valuable insight into the possibility of biofilm formation regulating pyoverdine, further work demonstrated the complexity of this regulation that was not interpretable from the screen alone. For instance, qRT-PCR of genes associated with pyoverdine biosynthesis and fluorescence microscopy of biofilm matrices suggested that ferripyoverdine uptake may be involved in biofilm-dependent regulation of pyoverdine production. These findings suggest that biofilm formation, a process which is not directly related to iron metabolism in the bacterium, utilizes iron-sensitive mechanisms to regulate pyoverdine production.

Furthermore, we were able to break the connection between biofilm synthesis and pyoverdine production in PA14, by either limiting iron with 1,10-phenanthroline or EDDHA, or by using low concentrations of the macromolecules that provide carbon and nitrogen. For instance, in nutrient-poor SK media, bacterial density is artificially restrained, which is likely to diminish cell aggregation and decrease production of quorum sensing molecules. M9 media, which is richer in nutrients, permits more robust growth. This difference may explain the variations in pyoverdine production and regulation that we observed. Our data indicate that the regulation of virulence factors like pyoverdine are highly complex, multifactorial, and are likely to take into account the conditions both within and outside of the bacterium.

Finally, we were also able to demonstrate that genetic or biochemical disruption of biofilm formation was sufficient to attenuate pyoverdine-mediated pathogenesis. This was an unexpected finding, as we had previously ruled out a role for biofilm formation in pyoverdine-mediated pathogenesis in the *C. elegans* liquid killing model (Kirienko et al., 2013). As proof of principle, we demonstrated that 2-amino-5,6-dimethylbenzimidazole, a small molecule inhibitor of biofilm formation, was effective in limiting *P. aeruginosa* virulence. This result also suggests a wider theme: disrupting any of the genes that we have shown to regulate pyoverdine production (including targets involved in motility, chemotaxis, signal transduction, or amino acid biosynthesis) may attenuate virulence without substantially compromising bacterial growth (which would place selective pressure on the pathogen to develop resistance). The interrelationships of virulence pathways demonstrate that it may be possible to compromise more than one pathogenic determinant with a single drug, which is particularly appealing.

The innate fluorescence of pyoverdine dramatically simplifies the process of identifying regulators of pyoverdine biosynthesis using high-throughput screening approaches; fluorescence is a nearly ideal readout for this type of screen. It should be admitted that regulators of virulence factors lacking ready detection techniques would be much harder to identify in this fashion. Arguably, the most effective method in these cases is to leverage model organisms to screen libraries (small molecule, transposon, RNAi, etc.) for pathogenesis; *C. elegans* and zebrafish are commonly used for this purpose (Begun et al., 2005; Miller and Neely, 2005; Garvis et al., 2009; Kizy and Neely, 2009; Feinbaum et al., 2012; Kirienko et al., 2016), because they are small, have rapid generation times, and exhibit strong evolutionary similarity to humans. This approach has been particularly successful in identifying drugs that might be repurposed to treat infectious diseases (Carvalho et al., 2011; Imperi et al., 2013; Kirienko et al., 2016; Kim et al., 2017). However, as noted above, this approach strictly requires that the virulence mechanisms, and their regulation, can be recapitulated in these models. If this demand can be met, this approach seems an invaluable resource for making discoveries ranging from the basic (e.g., the interconnectivity of regulatory networks that are involved in a variety of metabolic processes) to the clinical (i.e., the identification of therapeutics that can transform health care and postpone the rapidly approaching antimicrobial crisis).

## Author contributions

Conceptualization: DK and NK; Data Collection: DK; Data Analysis and Interpretation: DK and NK; Drafting: DK and NK; Critical Revision: DK and NK; Funding Acquisition: NK.

### Conflict of interest statement

The authors declare that the research was conducted in the absence of any commercial or financial relationships that could be construed as a potential conflict of interest.
