# Supplementary material for: High-Throughput Genetic Screen Reveals that Early Attachment and Biofilm Formation Are Necessary for Full Pyoverdine Production by Pseudomonas aeruginosa
Source: Front Microbiol. 2017 Sep 5;8:1707. doi: 10.3389/fmicb.2017.01707 (PMC5591869; doi:10.3389/fmicb.2017.01707)
Supplement: Supplementary file 1 [file Image1.PDF]

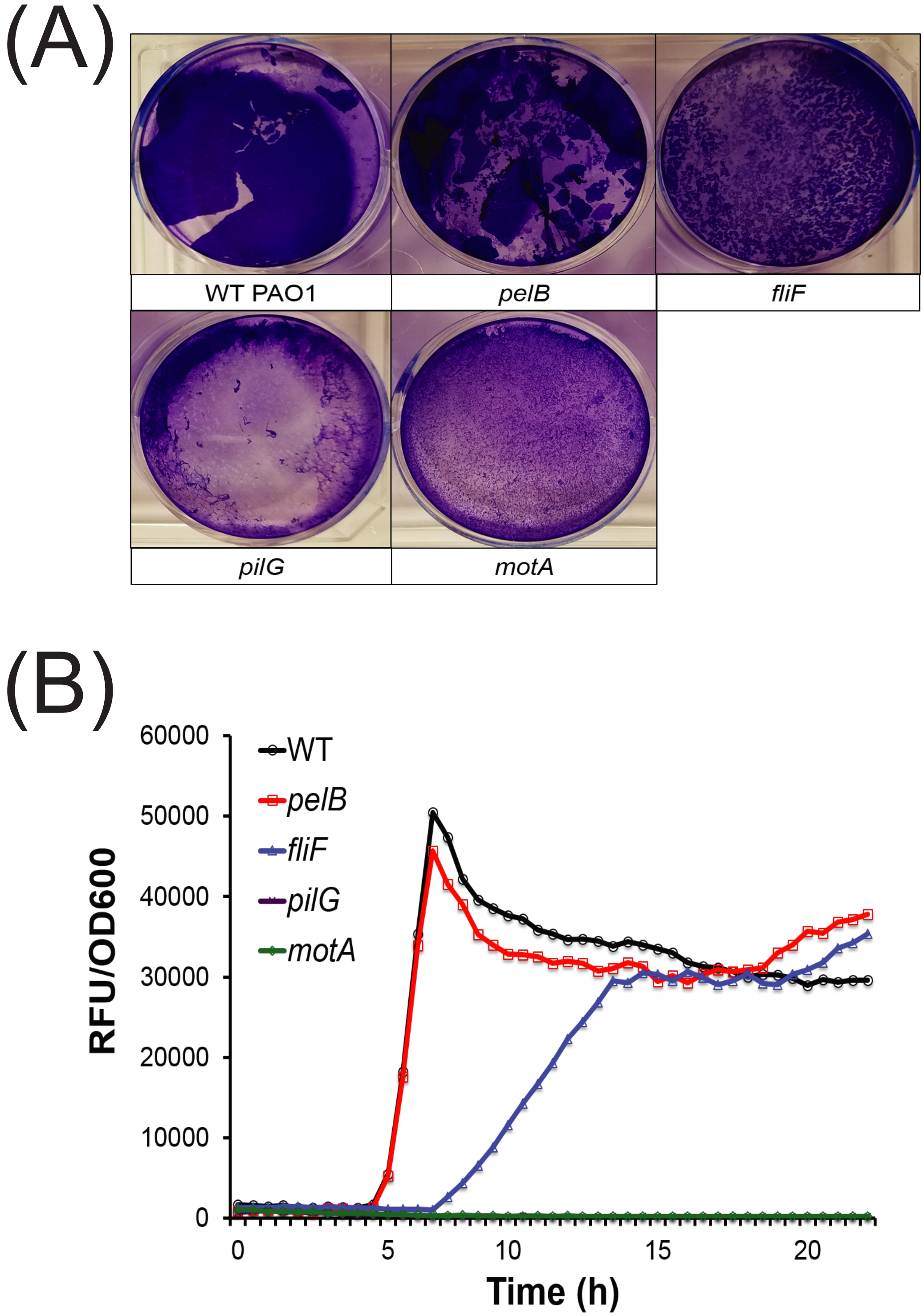

Supplementary Figure S1. Disruption of biofilm formation in *P. aeruginosa* PAO1 also significantly impairs pyoverdine production. (A) PAO1 biofilm mutants biofilm matrix in 6-well plate stained with 0.1% crystal violet solution. **(B)** Pyoverdine fluorescence normalized to bacterial growth kinetically measured over 24 h in PAO1 biofilm mutants. All data presented are representative results from three biological replicates. Pyoverdine production curves without bacterial growth normalization are available in **Supplementary Figure S6**.

Supplementary Figure S1
