# Supplementary material for: High-Throughput Genetic Screen Reveals that Early Attachment and Biofilm Formation Are Necessary for Full Pyoverdine Production by Pseudomonas aeruginosa
Source: Front Microbiol. 2017 Sep 5;8:1707. doi: 10.3389/fmicb.2017.01707 (PMC5591869; doi:10.3389/fmicb.2017.01707)
Supplement: Supplementary file 2 [file Image2.PDF]

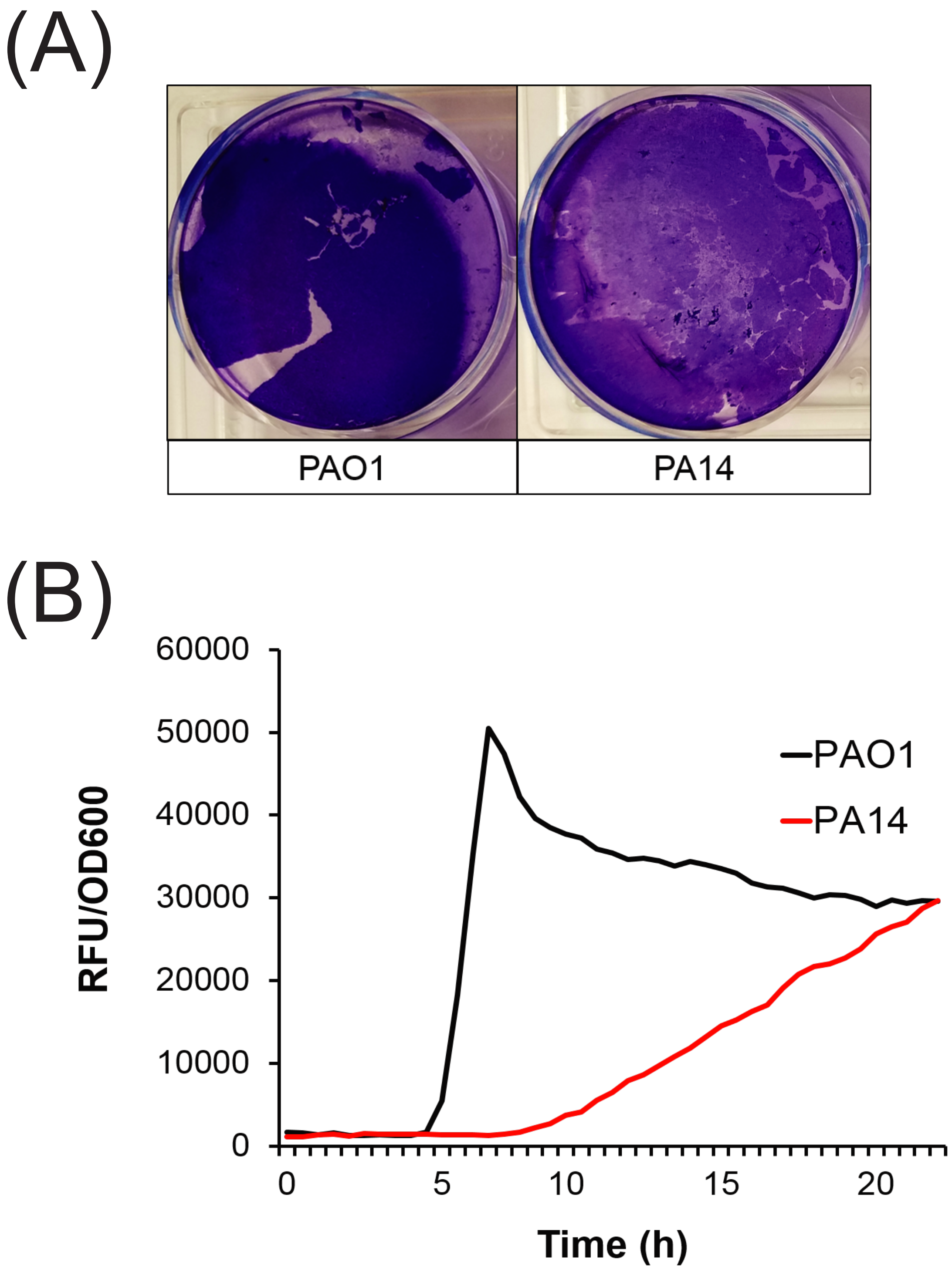

Supplementary Figure S2. Difference in biofilm formation between *P. aeruginosa* PA14 and PAO1 accounts for the difference in pyoverdine production. (A) *P. aeruginosa* PA14 and PAO1 biofilm matrix in 6-well plate stained with 0.1% crystal violet solution. **(B)** Pyoverdine fluorescence normalized to bacterial growth measured kinetically over 24 h in the two isolates. All data presented are representative results from three biological replicates. Pyoverdine production curves without bacterial growth normalization are available in **Supplementary Figure S6**.

Supplementary Figure S2
