# Supplementary material for: High-Throughput Genetic Screen Reveals that Early Attachment and Biofilm Formation Are Necessary for Full Pyoverdine Production by Pseudomonas aeruginosa
Source: Front Microbiol. 2017 Sep 5;8:1707. doi: 10.3389/fmicb.2017.01707 (PMC5591869; doi:10.3389/fmicb.2017.01707)
Supplement: Supplementary file 3 [file Image3.PDF]

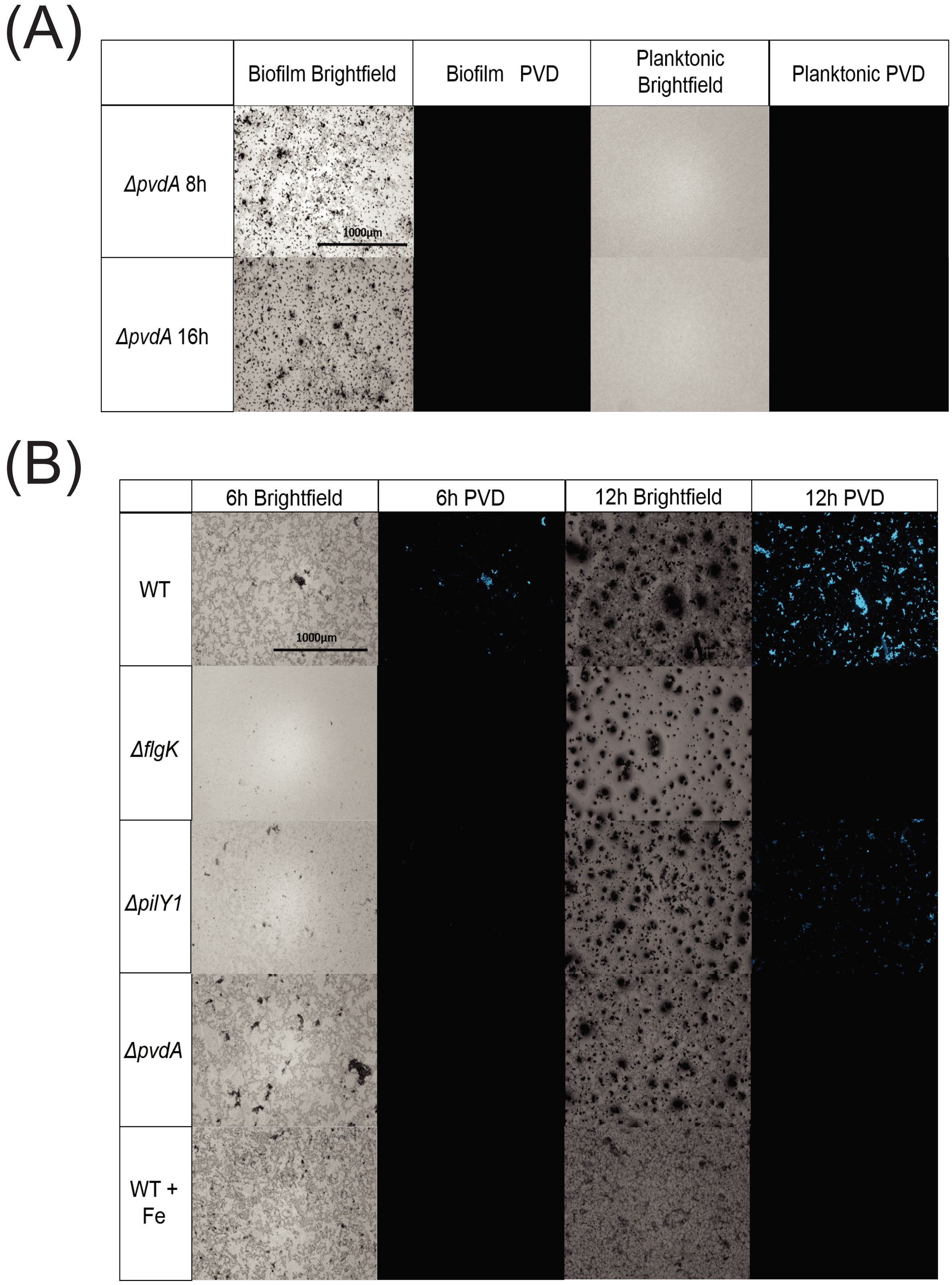

Supplementary Figure S3. (A) Pyoverdine production in PA14*ΔpvdA* biofilm matrix and planktonic cells imaged using pyoverdine-specific fluorescence filter. **(B)** Pyoverdine production in biofilm matrices of various PA14 biofilm mutants imaged using pyoverdine-specific fluorescence filter. All data presented are representative results from three biological replicates.

Supplementary Figure S3
