# Supplementary material for: High-Throughput Genetic Screen Reveals that Early Attachment and Biofilm Formation Are Necessary for Full Pyoverdine Production by Pseudomonas aeruginosa
Source: Front Microbiol. 2017 Sep 5;8:1707. doi: 10.3389/fmicb.2017.01707 (PMC5591869; doi:10.3389/fmicb.2017.01707)
Supplement: Supplementary file 4 [file Image4.PDF]

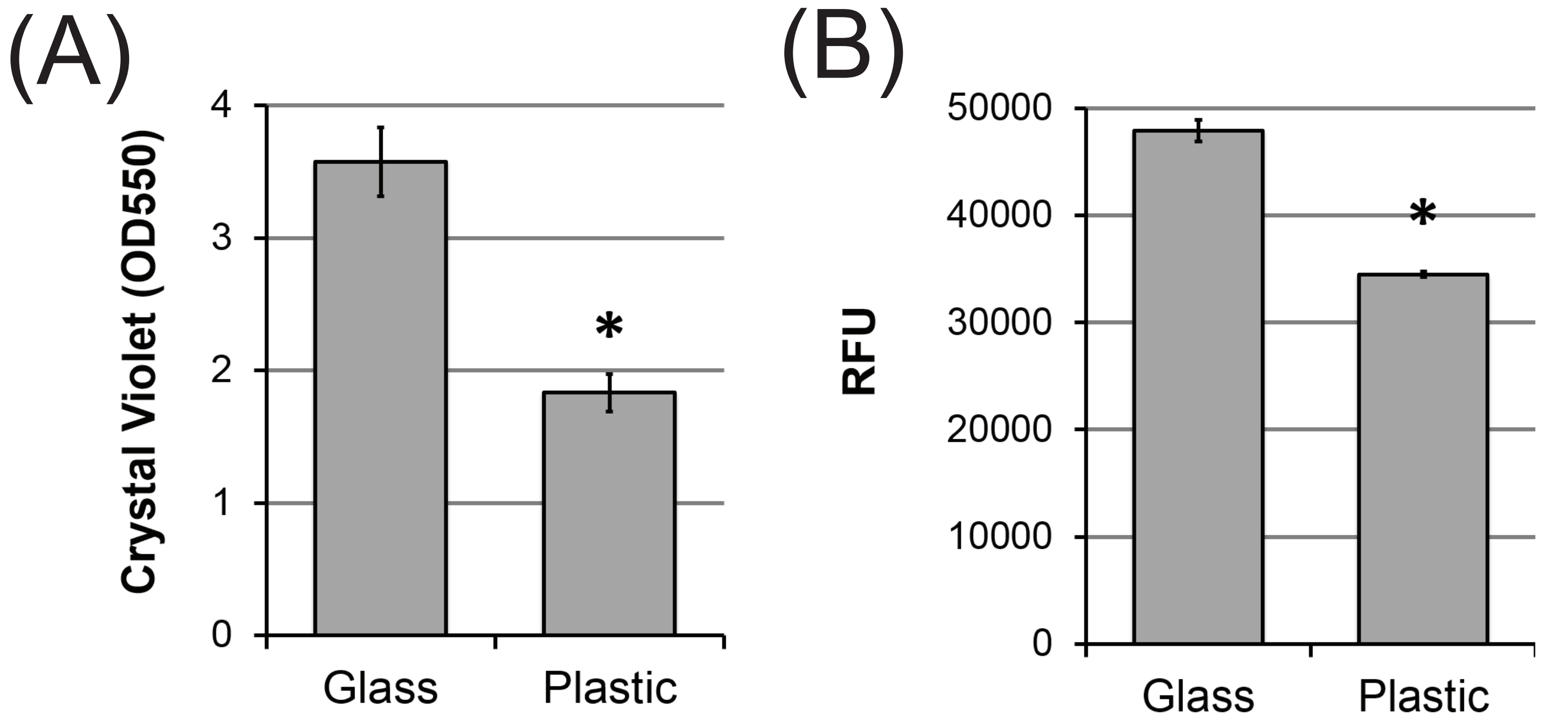

Supplementary Figure S4. Modification of attachment surface affects biofilm formation and subsequently pyoverdine production. (A) Quantification of crystal violet-stained biofilm matrix solubilized in acetic acid for *P. aeruginosa* PA14 grown on plastic or glass surfaces. **(B)** Pyoverdine fluorescence in bacterial supernatant after 16 h of static growth for PA14 grown on plastic or glass surfaces. All error bars in represent SEM between three technical replicates. Asterisks indicate significant difference between conditions (p-value < 0.01, based on Student's *t*-test).

Supplementary Figure S4
