# Supplementary material for: High-Throughput Genetic Screen Reveals that Early Attachment and Biofilm Formation Are Necessary for Full Pyoverdine Production by Pseudomonas aeruginosa
Source: Front Microbiol. 2017 Sep 5;8:1707. doi: 10.3389/fmicb.2017.01707 (PMC5591869; doi:10.3389/fmicb.2017.01707)
Supplement: Supplementary file 5 [file Image5.PDF]

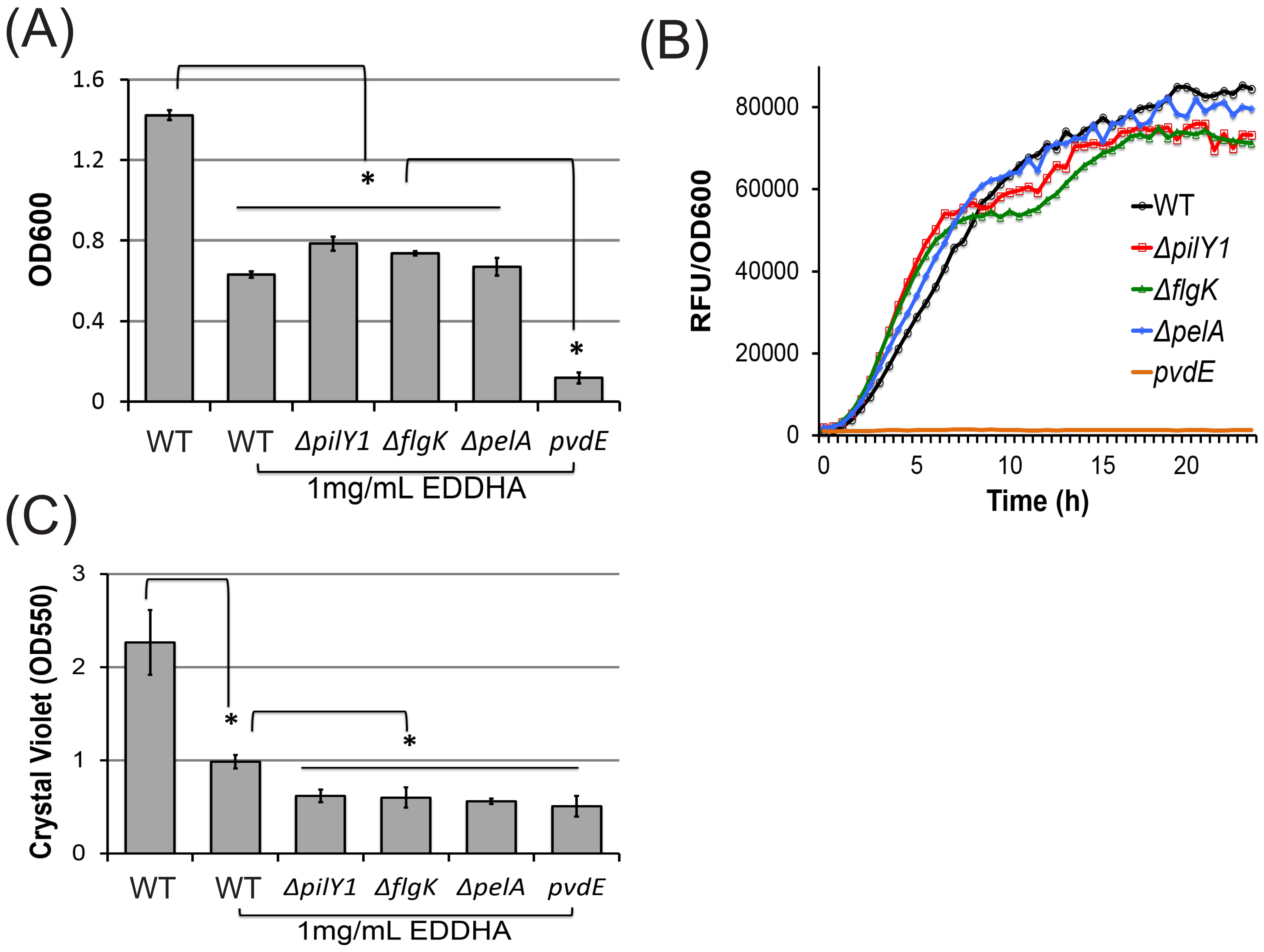

Supplementary Figure S5. Biofilm formation doesn't affect pyoverdine production under iron-starved conditions. (A) Bacterial growth of PA14 biofilm mutants grown in M9 media with 1mg/mL ethylenediamine-N,N'-bis(2-hydroxyphenylacetic acid) (EDDHA) ferric iron chelator measured by absorbance at 600nm after 24 h incubation at 30°C. **(B)** Pyoverdine fluorescence normalized to bacterial growth kinetically measured over 24 h in biofilm mutants grown in the presence of 1mg/mL EDDHA. **(C)** Quantification of crystal violet-stained biofilm matrix solubilized in acetic acid for PA14 biofilm mutants grown in M9 media with 1mg/mL EDDHA. All error bars represent SEM between three biological replicates. Asterisks indicate significant difference between conditions (p-value < 0.01, based on Student's *t*-test). Data presented in **(C)** is representative of results from three biological replicates.

Supplementary Figure S5
