# Supplementary material for: High-Throughput Genetic Screen Reveals that Early Attachment and Biofilm Formation Are Necessary for Full Pyoverdine Production by Pseudomonas aeruginosa
Source: Front Microbiol. 2017 Sep 5;8:1707. doi: 10.3389/fmicb.2017.01707 (PMC5591869; doi:10.3389/fmicb.2017.01707)
Supplement: Supplementary file 6 [file Image6.PDF]

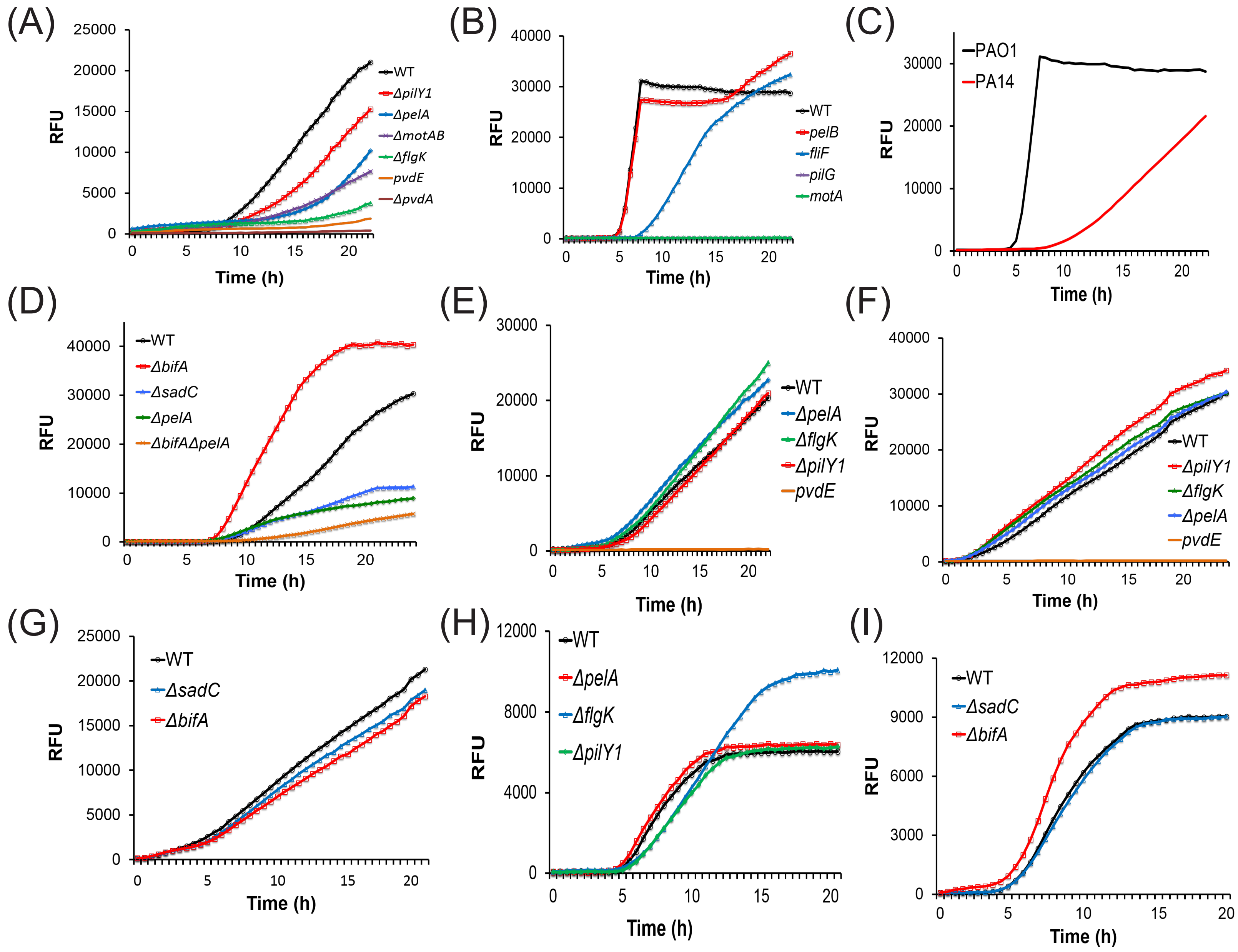

Supplementary Figure S6. Raw pyoverdine kinetics data without normalization to bacterial growth. **(A)** corresponds to **Figure 2B**, **(B)** corresponds to **Supplementary Figure S1B**, **(C)** corresponds to **Supplementary Figure S2B**, **(D)** corresponds to **Figure 4C**, **(E)** corresponds to **Figure 6B**, **(F)** corresponds to **Supplementary Figure S5C**, **(G)** corresponds to **Figure 6G**, **(H)** corresponds to **Figure 7A**, and **(I)** corresponds to **Figure 7D**. All data presented are representative results from three biological replicates.

Supplementary Figure S6
