# Supplementary material for: High-Throughput Genetic Screen Reveals that Early Attachment and Biofilm Formation Are Necessary for Full Pyoverdine Production by Pseudomonas aeruginosa
Source: Front Microbiol. 2017 Sep 5;8:1707. doi: 10.3389/fmicb.2017.01707 (PMC5591869; doi:10.3389/fmicb.2017.01707)
Supplement: Supplementary file 7 [file Table1.DOCX]

Supplementary Material

**High-Throughput Genetic Screen Reveals that Early Attachment and Biofilm Formation are Necessary for Full Pyoverdine Production by *Pseudomonas aeruginosa***

Donghoon Kang^1^, Natalia V. Kirienko^1*^

*** Correspondence:** Natalia V. Kirienko: kirienko@rice.edu

**Supplementary Table S1.** Full list of screen hits associated with biofilm formation. Hits that reoccurred are not listed.

| **Gene Name** | **PAO1 Homologue** | **Gene Function** |
| --- | --- | --- |
|  |  |  |
| **Initial Surface Contact** | | |
| *motA* | *PA4954* | chemotaxis protein |
| *motB* | *PA4953* | chemotaxis protein |
| *pctA* | *PA4309* | chemotactic transducer |
| *flgA* | *PA3350* | putative flagella basal body P-ring formation protein |
| *flgB* | *PA1077* | flagellar basal-body rod protein |
| *flgC* | *PA1078* | flagellar basal-body rod protein |
| *flgE* | *PA1080* | flagellar hook protein |
| *flgF* | *PA1081* | flagellar basal-body rod protein |
| *flgI* | *PA1084* | flagellar P-ring protein precursor |
| *flgJ* | *PA1085* | flagellar protein |
| *flgK* | *PA1086* | flagellar hook-associated protein 1 |
| *flgL* | *PA1087* | flagellar hook-associated protein type 3 |
| *flgM* | *PA3351* | putative negative regulator of flagellin synthesis |
| *flhA* | *PA1452* | flagellar biosynthesis protein |
| *fliA* | *PA1455* | motility sigma factor |
| *fliC* | *PA1092* | flagellin type B |
| *fliD* | *PA1094* | flagellar capping protein |
| *fliF* | *PA1101* | Flagella M-ring outer membrane protein precursor |
| *fliG* | *PA1102* | flagellar motor switch protein |
| *fliI* | *PA1104* | flagellum-specific ATP synthase |
| *fliJ* | *PA1105* | flagellar protein |
| *fliK* | *PA1441* | putative flagellar hook-length control protein |
| *fliL* | *PA1442* | putative flagellar protein |
| *fliM* | *PA1443* | flagellar motor switch protein |
| *fliN* | *PA1444* | flagellar motor switch protein |
| *fliP* | *PA1446* | flagellar biosynthetic protein |
| *fliQ* | *PA1447* | flagellar biosynthetic protein |
|  | *PA1103* | probable flagellar assembly protein |
|  |  |  |
| **Attachment and Adhesion Factors Production** | | |
| *pilB* | *PA4526* | type 4 fimbrial biogenesis protein |
| *pilC* | *PA4527* | type 4 fimbrial biogenesis protein |
| *pilF* | *PA3805* | type 4 fimbrial biogenesis protein |
| *pilR* | *PA4547* | two-component response regulator |
| *pilX* | *PA4553* | type IV pilus biogenesis protein |
| *pilZ* | *PA2960* | type 4 fimbrial biogenesis protein |
| *fimU* | *PA4550* | type 4 fimbrial biogenesis protein |
| *cupA1* | *PA2128* | fimbrial subunit CupA1 |
|  |  |  |
| **Biofilm Maturation** | | |
| *pelG* | *PA3058* | Pel exopolysaccharide transport protein |
|  |  |  |
| **Transcriptional Regulators that Affect Multiple Steps of Biofilm Formation** | | |
| *fleQ* | *PA1097* | Regulates flagellum motility and exopolysaccharide |
| *fleS* | *PA1098* | two-component sensor: regulates flagellum and adhesion factors |
| *fleR* | *PA1099* | two-component response regulator: regulates flagellum and adhesion factors |
